# Staff mental health while providing care to people with intellectual disability during the COVID‐19 pandemic

**DOI:** 10.1111/bld.12458

**Published:** 2022-02-08

**Authors:** Fintan Sheerin, Andrew P. Allen, Marianne Fallon, Philip McCallion, Mary McCarron, Niamh Mulryan, Yaohua Chen

**Affiliations:** ^1^ Trinity Centre for Ageing and Intellectual Disability, Trinity College Dublin Ireland; ^2^ School of Social Work, Temple University Philadelphia Pennsylvania USA; ^3^ Trinity Centre for Ageing and Intellectual Disability, Trinity College Dublin & Daughters of Charity Disability Support Services Dublin Ireland

**Keywords:** COVID‐19, intellectual disability, mental health, staff, stress

## Abstract

**Background:**

The COVID‐19 pandemic has placed enormous strain on health systems around the world, undermining the mental health and wellbeing of healthcare workers. Supporting people with intellectual disabilities may be particularly challenging for workers, as some people with intellectual disabilities may have a limited understanding of the pandemic, and find it challenging to adhere to the restrictions imposed by public health guidelines such as social distancing, lockdowns and change in usual routine and activities. In addition, many people with intellectual disabilities have increased vulnerability to more negative effects of COVID‐19, with significantly higher mortality rates. Although there is emerging research on the mental health of healthcare staff during this time, there has been little specific work on the mental health of staff working with people with intellectual disability, particularly a lack of qualitative research.

**Methods:**

The current study employed semi‐structured interviews with 13 healthcare workers (12 women and 1 man) who were working with people with intellectual disability during the COVID‐19 pandemic. The interview data were analysed using thematic content analysis.

**Findings:**

The participants spoke in depth about the challenges of the working environment, the impact of providing care during the pandemic on staff mental health, supporting staff mental health and wellbeing and learning for the future.

**Conclusions:**

Systematic efforts are required to protect the mental health of this staff cohort, as well as encouraging resilience and successful coping among staff themselves.

## Accessible summary

- COVID‐19 has changed the working environments of staff who support people with intellectual disability. Staff are feeling a lot of stress about this.

- Staff worry about giving COVID‐19 to people with intellectual disability or catching COVID‐19 from them.

- Other staff and managers can help staff to deal with this stress.

## 1 INTRODUCTION

In the aftermath of coronavirus in Wuhan, China, nearly 30% of nursing and medical staff reported moderate to severe mental health disturbance (Kang et al., 2020). The majority of research has focused on the well‐being and mental health of frontline healthcare workers in emergency and COVID‐19 wards. However, similar levels of stress may be experienced by healthcare workers in other settings, when working with people who may be particularly vulnerable to the infection and its sequelae.

A recent systematic review (Pappa et al., 2020) showed that healthcare workers working with vulnerable people are also experiencing many mental health challenges, such as stress, anxiety, depression, posttraumatic stress, insomnia, burnout and compassion fatigue. The most common risk factors reported were female gender, younger age, personal history such as a past psychiatric illness or being COVID positive, or limited professional experience. Each mental health challenge may also increase the risk for further mental health concerns, leading to a cycle of mental illness insults. In Rossi et al.'s (2020) and Luceno‐Moreno et al.'s (2020) studies, 19.8% and 79.3%, respectively of the participants were reported to have anxiety. A similar prevalence of stress was reported, from 21.9% (Rossi et al., 2020) to 74% (Maraqa et al., 2020). Fears were also very frequently reported, with 91.6% of interviewees fearing transmission of the virus to family, 87.7% worried that the disease has no treatment, and 85.8% concerned at acquiring the infection personally. The prevalence of depression ranged from 24.7% (Rossi et al., 2020) to 77.6% (Sahin et al., 2020). Insomnia was reported amongst 8.3% of participants in Rossi et al.'s (2020) study and in 50.4% in Şahin et al.'s (2020). HCWs in mental health care had higher levels of burnout and of hopelessness (37.5%) than those in nonpsychiatric multidisciplinary teams (24.02%) (Franza et al., 2020). In the United Kingdom's response to COVID‐19, National Health Service staff were offered mental health supports, with the key concerns identified including risk of infecting colleagues/family members, and medical violence (Dai et al., 2020).

The rapid onset of the COVID‐19 pandemic was met, in Ireland, by the announcement and introduction of restrictions on 12 March 2020, followed by full lockdown on 27 March (Government of Ireland, 2020). While this led to limitations on movement within general society (within a 2 km radius), it arguably resulted in much more stringent restrictions for people with intellectual disability, as they were deemed to be particularly vulnerable to infection and to severe consequences. These restrictions included the closure of day services, cessation of visits as well as impacting on family contact. This led to significant changes in the daily routines of people receiving service with a corresponding impact on staff patterns of working. It is unclear whether these changes led to mental health effects among HCWs in such settings, and, if so, how such effects compared to those in other health settings, as there is a dearth of literature in this regard. Maintaining continued quality of care for people with intellectual disability, with whom bonds have been formed over years of caregiving, has presented particular challenges during the current pandemic (Courtenay & Perera, 2020; Grier et al., 2020). While McCarron et al. (2020) has noted that many people with intellectual disability adhered to the restrictive measures, some may have difficulty in understanding or adhering to lockdown policies, and this may manifest through changes in their behaviours (Scheffers et al., 2021). In this context, staff are likely to experience particular challenges to their own mental health, which in turn may have the potential to adversely influence care for the persons with an intellectual disability they are responsible for and, indeed, the availability of frontline staff, should mental health concerns result in absences (Embregts et al., 2021).

Recently, two studies focused on healthcare workers working with people with intellectual disability (Lunsky et al., 2021). Both were online surveys, with either new, bespoke questionnaires or previously validated scales. One was performed in Canada, and the other in the United Kingdom. They reported a high level of anxiety and distress among healthcare workers. Poorer mental health demonstrated similar patterns as for other HCWs, such as fear of transmitting the virus or stress from the need for constant adaptability. There were also some specific patterns, such as the need to manage increased rates of mental health issues and aggression of the people they support. Online surveys offer certain advantages but are often limited by the closed nature of their structured questions (Andrade, 2020). A third study qualitatively examined the experiences of direct support staff, and documented similar concerns, but also pointed out examples of successful coping and the desire of staff for support from other professional and peer groups (Embregts et al., 2021).

It is important to acknowledge the effect of COVID‐19 and associated restrictions on the mental health and wellbeing of people with intellectual disabilities, themselves. Recent evidence has emerged from the Intellectual Disability Supplement to the Irish Longitudinal Study on Ageing (IDS‐TILDA) reporting increased stress and anxiety related to not being able to attend work/day service, lack of contact with families and friends, and loneliness (McCarron et al., 2020); findings corroborated by T. Murphy et al. (2020).

The aim of this study was to understand the impact of COVID‐19 and related restrictions on the mental health of staff providing care to people with intellectual disabilities, during the pandemic in Ireland. Furthermore, it sought to explore staff experiences in engaging with mental health support, types of assistance needed and experienced as helpful in reducing any related mental health concerns and increasing preparedness to continue to provide ongoing care to people with intellectual disability and, finally, challenges and opportunities for supporting staff mental health into the future.

## 2 METHODS

### 2.1 Design

A qualitative descriptive design (Sandelowski, 2010) was employed to address the research aim through the conduct of individual interviews with participants.

### 2.2 Sample

Participants in the study were health and social care workers who were working with people with intellectual disability, in three Irish intellectual disability services, during the COVID‐19 pandemic in Ireland. The definition of health and social care workers here was broad and included those who had daily or intermittent care interactions with people with intellectual disabilities (e.g., social care workers, care staff, registered intellectual disability nurses, health care assistants, care staff and clinical/unit managers, and other professionals with frequent direct care interactions such as multidisciplinary team members). A letter of invitation was sent to the senior management of the three convenience‐sampled disability service providers, requesting access and distribution of study information to staff. All three services were located in the eastern part of Ireland. Two reminders were sent. Each service decided on their own best channels to communicate with their staff, which were usually announcements via intranet, or emails. Once potential participants approached the research team, they were provided with the participant information leaflet and consent form. Once participants returned a signed consent form, the research team arranged a time for the interview.

As this study employed a qualitative methodology, a power analysis was not appropriate. Choosing a suitable sample size in qualitative research remains an area of debate and uncertainty (Vasileiou et al., 2018). Nonetheless, we recognised the importance of obtaining interviews from a range of healthcare staff working with people with intellectual disability. The data was interrogated to a sufficient degree to identify all key factors relating to health and social care workers' mental health, as raised by the participants.

### 2.3 Data collection

Data were collected through the conduct of individual interviews (online and telephone due to COVID‐19 restrictions), each a maximum of 60 min duration. A semi‐structured interview protocol was developed, based on literature, with a focus on the dynamics of mental health and coping strategies, as well as broader factors that may support or pose challenges to mental health in this staff cohort. The protocol probed:

1. COVID‐19 related antecedents, beliefs and factors and their effects, if any, on staff mental health.
2. How any changes in staff mental health affected their ability to provide quality care.
3. Descriptions of specific mental health needs/supports.
4. Barriers and opportunities for engagement with mental health supports.
5. Challenges, opportunities and support requirements for meeting staff mental health needs.

### 2.4 Analysis

Remote interviews were recorded and transcribed, pseudonymized, and resulting data were analysed using thematic analysis (Braun & Clarke, 2006). Initially, an iterative analysis process was used to generate codes and identify themes within the interview data by three members of the research team (authors YC, APA and FS). Given the breadth and novelty of the research questions, and the flexibility of the semi‐structured interview format, a data‐driven approach to analysis was used to suggest themes that may not have been explicitly addressed in the questions. As a next formal step these themes were discussed with another member of the research team and refined as necessary.

### 2.5 Ethics

All ethical principles were adhered to and ethical approval was received from the National Research Ethics Committee for COVID‐19‐related Health Research (NREC COVID‐19), approval number 20‐NREC‐COV‐068. A data protection impact assessment was undertaken and approved in line with the General Data Protection Regulation.

## 3 FINDINGS

Thirteen interviews were conducted by YC and FS over the period November 2020 to January 2021 with a predominantly female sample of self‐selecting individuals who provided healthcare to people with intellectual disability during the pandemic (Table 1). These interviews lasted approximately 30 min each.

**Table 1:** Participant demographics

	Number of respondents
Gender	
Women	12 (92.3%)
Men	1 (7.7%)
Age group (years old)	
30–40	6 (46.2%)
40–50	6 (46.2%)
>50	1 (0.7%)
Role	
Direct support workers (social care, care staff)	2 (15.4%)
Behaviour specialist	1 (7.7%)
Physiotherapist	1 (7.7%)
Clinical nurse specialist (acute care)	1 (7.7%)
Managers (nursing, social care and area management)	8 (61.5%)

**Figure 1:**
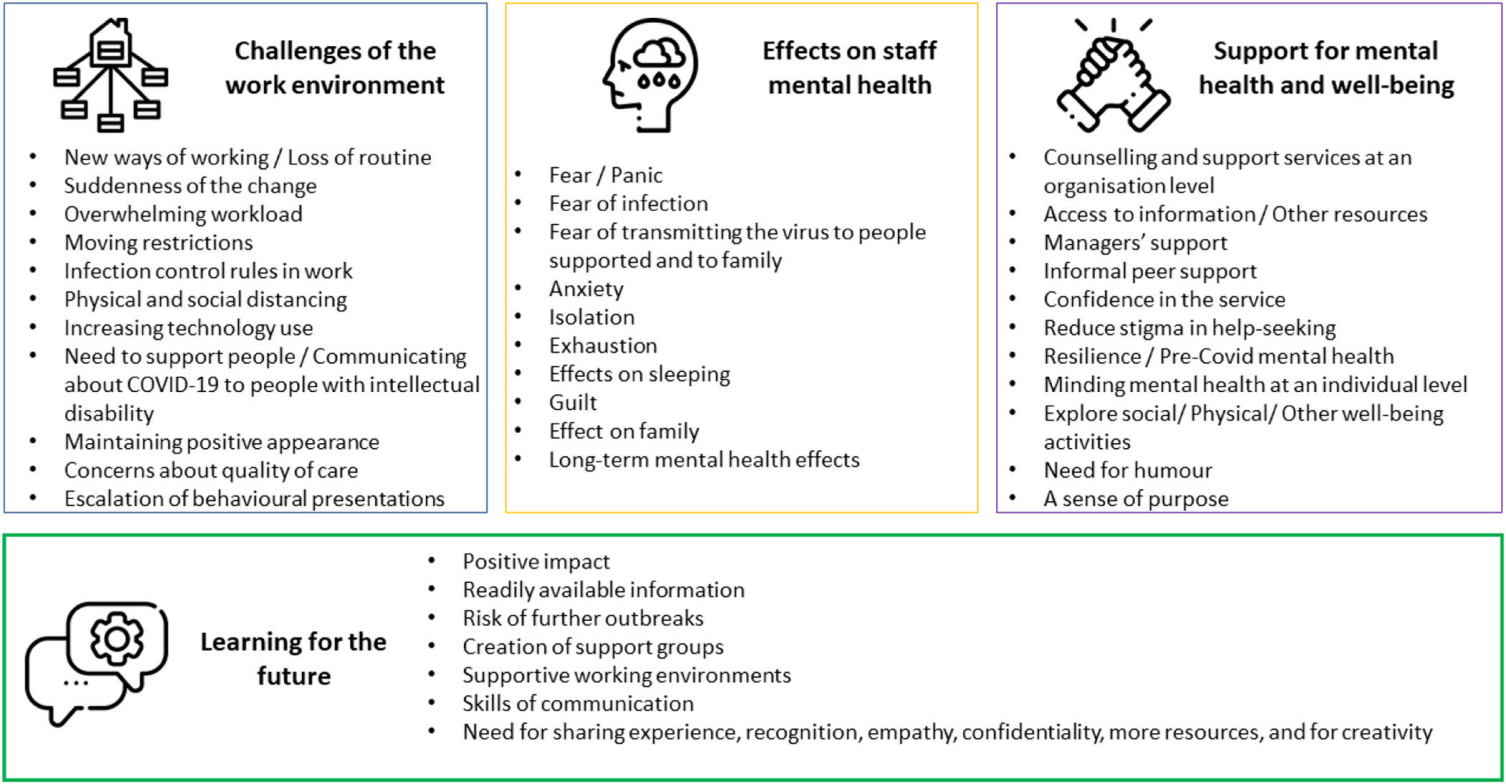
Themes identified [Color figure can be viewed at wileyonlinelibrary.com]

Four main themes emerged from the analysis of the interview narratives (Figure 1). These related to the challenges of what had been a changing service environment, the effects of care during the pandemic on staff mental health, what participants found supportive in respect of their mental health and wellbeing and lessons for the future. These will now be presented.

### 3.1 Challenges of the work environment

The abrupt nature of the changes brought on by the pandemic‐related restrictions was highlighted, with some participants indicating that staff did not have sufficient time to prepare themselves for the pervasive changes they were about to face,

*…for a lot of the people using our services, all of a sudden, basically overnight, people were no longer able to go to their regular jobs, their work experience* [and] *to their day service setting*. (Petra)

These stringent restrictions, brought on by public health guidelines, resulted in major changes in care—and work—environments, with new ways of working and new shift patterns. It was noted that this was a period of unease and one of major adjustment. Amongst other things, new infection control procedures were introduced with COVID testing and the employment of personal protective equipment (PPE). Accessing these was stressful,

*…difficult access to the appropriate PPE, and to the COVID testing was also stressful*. (Zara)

There was also a need to spend substantial time cleaning, thus affecting the amount of time available for other duties.

*The cleaning procedures are heightened so staff are spending a lot more time* [on it]. (Caroline)

Physical distancing also became the norm, and this posed difficulties for staff working with people with an intellectual disability, as it was difficult to explain the necessity for such distancing to those who often wished to have greater physical closeness to staff. This was anathema to how most staff would have wished to conduct their interactions,

*So, you are like running away from them… you know; don't come near me*. (Fabiana)

*…some of them at the very start couldn't understand why they had to stay back from us*. (Gabrielle)

This, and the movement of staff and people with intellectual disability across service sites, led to the breakup of staff teams and those who received their services, something that was noted by participants,

*…we are not altogether as a team like we normally were in the one area… we are now spread out over a community with different houses supporting people with intellectual disabilities*. (Fabiana)

*…you had a new manager, you had a new staff team, you had new service users and you also had a roster which you never had before*. (Na)

This likely contributed to feelings of unease, but was accompanied by changes to working patterns that resulted in staff having an increased workload and with a regular need to work well beyond normal working hours,

*We are working outside of our paid hours, like I never finish my work on time never you know*. (Ger)

Many of these problems had their origin in reduced staffing levels, related to illness and exposure to COVID‐19; there were less staff available for the enhanced workload. This created a vicious circle, with excessive workloads and unpredictable changes in work fuelling absences that further contributed to that excessive workload,

*…you might have x, y and z supports but you don't have the staffing levels*. (Gabrielle)

*…we have lost staff along the way who just haven't been able to cope with it*. (Petra)

As time passed, procedures and processes became more routine, which had the effect of reducing the anxiety brought about by the onset of the pandemic and related restrictions. Participants noted that a sense of stoicism emerged, as people sought to be positive while, at the same time, *surviving*; suggesting they were just getting by.

*Well now it's six months down the line, everyone is very used to the restrictions regarding COVID‐19 like social distancing everyone wears their face masks, you know, everyone is kind of used to the whole procedure around it*. (Sadhbh)

*… what I feel that I am doing is I am just getting used to it and surviving*. (Dominique)

In light of the increased workload, perceived professional commitment and associated willingness to push on with a brave face may have left staff vulnerable to mental health concerns,

*…we don't have a choice I mean you are a nurse; you have to be resilient do you know so, (laughs) you just have to work professionally, you know?* (Dominique)

Participants noted that they continued to work in this way against a backdrop of needing to balance concerns and expectations. Their first concern was for the health and wellbeing of the people whom they supported, and this was imbued with the sense that there were expectations to maintain routine for people, despite the dynamic challenges to that routine across the services. Thus, there was concern about the inability to meet expectations,

*…it's a really important thing that they have regular staff supporting them; staff that know them*. (Francesca)

They were also concerned that any non‐adherence to restrictions could potentially spread the disease among people with an intellectual disability, leading to fear and, when a staff member was infected, to guilt,

*…we were all very concerned that we would be bringing something into people's homes where we were doing the testing*. (Zara)

The reopening of some service areas following the initial wave (in August 2020), were highlighted as being not only good for people with intellectual disabilities but also for staff mental health,

*…it still is better that the day services are open and all that for the guys and for, I will say this, for staff mental health*. (Gabrielle)

These services did not, however, remain open as Ireland was hit by a second COVID‐19 wave from August to November 2020, with a further, and particularly challenging, third wave leading to a prolonged lockdown from the beginning of January 2021.

### 3.2 Effects of pandemic on mental health of staff

The sudden and prolonged nature of the COVID‐19 restrictions, and their effect on intellectual disability service provision, led to clearly described mental health and wellbeing challenges for staff, with fear and negative thoughts manifesting in disrupted sleep and constant tiredness. This was compounded by the more generalised effects of societal restrictions on staff members' families and life patterns.

The immediate response to the pandemic has been alluded to above and many participants used the word *panic* to describe this,

*…at the start…everyone was in a panic because we didn't really know what to expect*. (Francesca)

Intellectual disability service provision is, by its nature, relatively predictable, with recognisable patterns and manageable phenomena. The sudden COVID‐19 related service changes removed this, leading to insecurity and associated stress whether a staff member was working remotely or if they were providing hands‐on care. For some participants, change meant having to work from home. One behaviour therapist identified that this brought a feeling of isolation,

*…nearly everybody was… finding it very stressful… it was driving them mad working from home… not meeting anybody*. (Mary)

Others providing hands‐on care found that the need to remain for long hours in a set service living unit, with physical distancing, also led to stress related to isolation,

*…a feeling of isolation to be honest there is so much less interaction not only with the people we support but also with my peers and co‐workers*. (Zara)

The fear of infection was also a cause of anxiety for some participants, who were concerned that they might have been exposed to COVID‐19,

*…I know that they* [colleagues] *have this underlying anxiety and fear that they are going to catch something that it is going to be devastating*. (Zara)

It was noted that staff were concerned about the health of those whom they supported, and the risks associated with them becoming infected. This was another source of anxiety,

*…they were very anxious about people they were supporting who may have contracted COVID…* (Mary)

This fear led to some participants constantly questioning themselves and worrying that they might have done something that could impact on the health of the people with intellectual disability,

*…there is a level of guilt that you are not doing every single thing, all the time, for everyone that you are being requested to do*. (Zara)

Constant stress and anxiety can have significant effects on one's health and this can be manifested in altered daily activities, such as not sleeping despite being exhausted,

*…you are actually exhausted, do you know? You are wrecked because it does have a big impact on you and your energy levels like*. (Fabiana)

Despite being *so tired you just wanted to go to bed* (Ger), exhaustion did not bring sleep,

…[staff] *would go home from work and have maybe sleepless nights worrying about outcomes of…people who were coming back positive… how …their health was going to be during it…* (Sadhbh)

…and any sleep achieved was far from restful,

*I have had some more like had some COVID dreams… like anxiety dreams… my sleep was not as good; I was waking up in the middle of the night…* (Ger)

Alongside this were the myriad of other issues visited on citizens by the pandemic and restrictions, related to childcare, home‐schooling, job‐loss and caregiving. Participants spoke of how this further contributed to exhaustion and the feeling that time spent at home *is not a break* (Zara).

A number of participants pondered what the long‐term effects of caring during the pandemic would have on the mental health and wellbeing of staff, particularly those with underlying mental health concerns,

*I do suffer from mental health problems. I'm on medication for it and since the pandemic I'm at the maximum level of my current medication*. (Gabrielle)

It was noted that there would be a need to address these issues as society moves forward,

*I don't think we've really tackled the mental health side at the moment. I think we are still in fight mode. I'm not sure that we've started to do that reflection piece and healing because we are still in the wound*. (Caroline)

### 3.3 Supporting the mental health and wellbeing of staff

The participants reported a range of solutions to support mental health and well‐being in this challenging period, provided by their institutions, or from their personal resources. A number of organisations had extant employee assistance programmes (EAPs) in action, which were available to staff at any time,

*…we have an EAP programme within the service like you can report a COVID‐19 so if you had trouble at home or if you were deeply traumatised by a situation at work or if you had a bereavement or whatever…* (Fabiana)

These programmes were supplemented by other diverse supports (telephone counselling, one‐to‐one supervision with managers, mindfulness training, for e.g.),

*…as* [clinical nurse] *managers… if any team has been through a stressful time, we will do one‐to‐one supervision sessions*. (Petra)

*…there was supports… for workers as well mindfulness kind of activities and things like this and that was really invaluable*. (Ger)

Other sources of support included, for example, Health Service Executive (HSE) services, individuals' own private health insurance providers, and general practitioners,

*We've always had the* [private health insurer] *outside, the external helpline we can ring that's always in place 24 hours seven days a week*. (Sadhbh)

*…well, I actually reached out to the HSE service, what is it, you got four complementary one‐to‐one counselling sessions. So, I availed of that*. (Na)

Informal support from managers or from colleagues was reported by the participants as the cornerstone to building well‐being, especially when processes moved online. These informal supports were positively assessed by the participants and considered more useful than formal supports. This appeared to be grounded in the personal knowledge that colleagues had of each other,

*…my colleagues and myself would be quite supportive of ourselves if we notice the one person that was kind of struggling*. (Sadhbh)

Participants reported that having access to accurate and appropriate levels of information helped to reduce anxiety and thus contribute to positive mental health and wellbeing. Hence, having access to reliable information about the pandemic was indeed helpful in supporting mental health,

*…we were getting an update every week, so like the management are great how they updated us*. (Mary)

One participant noted, however, the importance of finding a balance in this regard, as she felt that the amount of information could be overwhelming for the staff,

*…there was in my opinion an awful lot of misinformation and a mixture of say… over‐information as well as in almost telling us too much*. (Gabrielle)

It was also considered important that institutions offer proper protection from COVID‐19 to their staff; this helped to foster a relationship of trust,

*…they know that they are OK in work because they have the PPE gear*. (Francesca)

*…the service is really good in making sure that the contract tracing is up to the standards*. (Dominique)

Staff also drew on their own personal resources to protect their mental health and wellbeing. It has been noted that collegial support was important in this regard, but some felt embarrassed to talk,

*…I would talk about like to my team that I have accessed support there is no shame in it*. (Chris)

*Oh yes! It's definitely an embarrassment thing or maybe they feel weak or oh my god if someone knew I needed help it's embarrassing*. (Ger)

Other solutions to supporting oneself were identified, and these were individually determined. These included mindfulness, online social activities, listening to music, exercise and meditation. Physical activities, and especially outdoor activities were the most common activities mentioned by participants, even when the distance allowed was short,

*You know that we are getting out for our walks and we are you know contacting people remotely with zoom and other things you know so I have been quite involved in voluntary stuff as well*. (Ger)

Altruism was evident in the contributions of participants. Unfortunately, this altruism can lead to staff neglecting their own well‐being. With this in mind, participants often mentioned the importance of protecting mental health at an individual level and prioritising this in what was an overwhelming context,

*…I had to prioritise; I only had so much time to give and what… You do have to strike a balance it has been difficult I think trying to be creative how we can reduce the isolation I think it's making an effort to check in with people in my department*. (Zara)

The general restrictions and the work changes led to social isolation with feelings of loss. Thus, social activities, albeit virtual, were cited by many of the participants as playing a compensatory role in this regard.

*I suppose work was great because it was very sociable even if it was on Zoom and that and I didn't start working remotely straight away in March because the software wasn't set up*. (Mary)

Finally, being able to maintain a sense of humour and a positive perspective was also considered to be important in protecting mental health.

*Because we've to make things fun for the guys* [people supported] *and we are like what makes us happy might make them happy might not we can try it*. (Gabrielle)

### 3.4 Learning for the future

This study primarily focused on how staff mental health and wellbeing was impacted while caring for people with intellectual disability during the COVID‐19 pandemic. Despite this, many participants wanted to speak about future needs, both in terms of how the state and services might respond to future health crises and support the mental health needs of staff who were negatively affected during this pandemic (Figure 1),

*You know, I think it's only when the event is over, and we can all kind of look back and go ‘oh my God you know that was so difficult’. I think that is where we will really see the effects of what we are going through at the moment…* (Petra)

The importance of peer support and space for talking was particularly noted, with a consideration that the *natural understanding peers have for one another* would be facilitative,

*…when you actually have an opportunity to share experiences and learn from experiences it allows an opportunity to support one another…* (Zara)

Because of the caring nature of their position, one interviewee also suggested that the positive support from peers should be better valued,

***… **But I think there is certain like there is it's a natural fit that the work we do is about caring but that we can care for each other as co‐workers you know that is what I am saying there is ways we could be a bit more conscious about it*. (Ger)

One manager identified the potential for the development of positive supportive environments for staff,

*I was thinking of creating at my workplace a space for people to talk, to show the recreational things that they do, whether they like fitness or whether that's being part of a choir you know in a cycling club or whatever*. (Ger)

It was acknowledged that there is sometimes a fear or even stigma about opening up to others and that supportive environments might facilitate this,

*…sometimes people are afraid to admit that they are stressed or anxious about a situation. They don't want their manager to think they are not coping… maybe more awareness for them to not to be OK*. (Sadhbh)

As mentioned in the first theme, the suddenness of the change and the uncertainty were challenging for staff. Many suggested having accessible and timely information for the future,

*…So definitely, I think I need to have more information on that, you know for somebody not to have to go looking for it*. (Mary)

The management approaches should be more proactive and supportive, along with the development of communication skills,

… *I think it's very important that you don't have to go to a liaison person… that you can just see it online and you can avail of it anonymously*. (Mary)

Participants reported many useful suggestions to better prepare for further outbreaks especially for staff working in the area of intellectual disability, including the need to increase awareness among the general public, to get more recognition for staff, to get more resources,

*I wish the general public would be more responsible (*laughs*) you know as a health care worker I think there needs to be a lot more education even in schools now that all the young people are back in school*. (Zara)

Unexpected positive impacts have also been experienced by some of our participants, such as the time saving with the remote connection,

*But there is definitely merit in it that if we want to meet with our colleagues forty miles up the road, we don't have to be wasting three hours driving we can do it through different mediums. So, it has probably made us become more efficient*. (Petra)

This unprecedented episode also allowed people to enjoy again linking with families or reflection time,

*I suppose really build up relationships with families because we don't really need to link with families unless something changes*. (Petra)

As for the institutional support for mental health, one interviewee emphasised the importance of their sustainability,

*I think mental health supports should be there in the organisation all the time. Just you know a pandemic or no pandemic*. (GER)

## 4 DISCUSSION

Our study analysed thirteen interviews over the period November 2020 to January 2021 with predominantly female participants, who were health and allied health professionals/social carers/managers working with people with intellectual disability. We identified four main themes: (1) Challenges of the work environment; (2) effects of care on staff mental health; (3) support for mental health and well‐being; and (4) learning for the future.

Staff working with people with intellectual disability reported similar patterns of mental health challenges to those reported for health and social care workers in other healthcare settings, such as acute hospitals, day centres, and longer‐term care areas, for example, nursing homes (Luceno‐Moreno et al., 2020; Pappa et al., 2020; Rossi et al., 2020).

The abrupt change in the working environment led to major changes in the care and work environment. The constant adjustment has been reported as overwhelming for health and social care workers in general. In a structured online survey, behaviour health care providers also pointed out that all of the changes and challenges left staff feeling burned out and overworked (A. A. Murphy et al., 2020). The rapid changes were particularly difficult due to this succession of waves. In our study, staff felt tired because of the difficulty in taking leave or the need to work extra hours. The situation of irregular working hours was also observed in another online survey among nurses (Alshekaili et al., 2020). In a study of psychotherapists providing support during the pandemic, it has also been reported that 74% of such staff were tired (Aafjes‐Van Doorn et al., 2020), leading to the cycle of stress proposed above based on Pappa et al.'s (2020) work.

The high level of change and the corresponding increase in expectations were very challenging for staff in our study. A similar level of frustration and concern about not meeting expectations has been expressed by health care workerss working with older people with cancer (Krok‐Schoen et al., 2020).

In respect of staff mental health, participants often reported panic, anxiety and fear, similar to the reports of anxiety in other studies (Luceño‐Moreno et al., 2020; Maraqa et al., 2020; Rossi et al., 2020). Participants also reported insomnia, something seen in Şahin et al.'s (2020) study.

A variety of supports were reported in our study. Usually, informal peer support, proactive managers' support, compensating activities and access to formal counselling supports (such as EAPs offered by employers) were noted. These protective factors were also identified in other studies (Maraqa et al., 2020; Nowicki et al., 2020).

Positive impacts of this pandemic on practice were also identified in our study, reminiscent of a Polish online survey among nurses, which identified opportunities brought about by the pandemic for improving practice (Nowicki et al., 2020). The participants in the current study also offered a variety of suggestions for the future. However, the negative impact on service organisation and provision was significant, given the closure of so many settings. It is noteworthy that most of the studies pertaining to the mental health of health and social care workers were based in clinical hospital contexts, where high levels of COVID‐19 were seen. These were mainly “front‐line” staff (Dai et al., 2020; Luceño‐Moreno et al., 2020; Pappa et al., 2020; Rossi et al., 2020; Sahin et al., 2020) or were proving care to patients with acute/chronic illness (Krok‐Schoen et al., 2020). The nature of the participants' roles in the current study was such that their experience of burden was likely different to that of health and social care workers in those other settings, although we did not compare these different cohorts of health and social care workers within this study.

The participants in this current study were required to be constantly creative, to deal with the restrictions, and the increased incidence of mental health issues or behavioural disorders, which was reported by another survey among staff with people with intellectual disability (Lunsky et al., 2021), provided particular complexity to the caring environment. Unlike some other groups of adult care recipients, staff had particular concerns regarding the potential nonadherence of people with intellectual disability to restrictive measures; this contributed to the burden of care and stress experienced by participants. The staff here were not alone in this concern given reports of heightened neuropsychiatric symptoms of dementia (Simonetti et al., 2020) and reports for many groups of increased stress during COVID‐19 (Cohen et al., 2020). For staff working with people with intellectual disabilities the challenge may also have been that they view their work as increasing not restricting opportunities inherent in the philosophy of service (Health Care Executive, 2011).

As previously mentioned, coping strategies employed by participants included peer support from and with their colleagues, clinical supervision with managers and availing of formal mental health supports at the workplace. Clinical supervision has previously been reported as helping reduce burnout among staff working with people with intellectual disabilities and other disabilities (Gibson et al., 2009). Resilience was evident in the participants' humour, sense of purpose, and amongst those whose experienced positive mental health before the onset of the pandemic. Conversely, those with pre‐existing mental health issues before the COVID‐19 pandemic are likely to require a greater level of support, based on feedback from participants in this study. For staff with pre‐existing mental health issues, more proactive efforts to support their mental health may be useful, such as reminders of the supports available at work, and through active peer support.

## 5 LIMITATIONS

We designed the study to allow an open and safe space for participants to discuss their experiences and perspectives. A methodology using predetermined questions in an online survey might have been quicker and have reached a wider sample, but it would also have lost the richness of qualitative data gathered in this study. As the participants were self‐selecting, we might have missed responses from people who felt less comfortable talking about their mental health. We tried to overcome this limit by inviting participants to talk about staff mental health during COVID‐19 in general terms, relating it to themselves when they felt comfortable to do so. Our study interviewed each participant at a single timepoint, and thus it did not track changes in stress levels, mental health and coping strategies over the duration of the pandemic. Some of the interviews were relatively short compared to others; however, using experienced interviewers and a semi‐structured interview schedule ensured there were follow‐up questions, and prompts to add further points to help ensure that interviews did not end before respondents had made the points they wished.

While studies are beginning to provide qualitative insights into the impact of the COVID‐19 pandemic on mental health of staff working with people with intellectual disability (Embregts et al., 2021), this is the first on to do so in an Irish context.

## 6 CONCLUSION

Staff working with people with intellectual disability suffer from the same work overload, the sudden change, the constant adaptation, and the impact of the pandemic on their mental health, as other health and social care workers. Due to the specific conditions of their work, and the needs of those whom they are supporting, the impact of the pandemic appears to be significant with solutions needed to address the mental health of health and social care workers across personal, institutional and policy levels.

At an individual level, coping strategies should be encouraged such as availing of support from peers and managers, as well as availing of more structured mental health supports. At an institutional level, appropriate levels of timely information about COVID‐19 and available supports should be provided to staff, with clear communication between staff directly providing care and management staff. At a broader level, sufficient resources should be made available for PPE, staff mental health support, and staffing levels more generally, to avoid burnout associated with working excessive hours. At time of writing, lockdown restrictions are easing in Ireland, with very high uptake of COVID‐19 vaccinations. Nonetheless, as one of our participants noted, the full mental health impact of this pandemic may not be fully known for some time to come.

In summary, the study highlighted the substantial challenges experienced during the COVID‐19 pandemic, the impact of providing care on staff carer mental health, and the importance of supporting staff through developing a positive and supportive work environment that in turn better supported people with intellectual disability.

## Data Availability

The data that support the findings of this study are available from the corresponding author upon reasonable request.
